# Supplementary material for: Homologous Recombination Deficiency and Cyclin E1 Amplification Are Correlated with Immune Cell Infiltration and Survival in High-Grade Serous Ovarian Cancer
Source: Cancers (Basel). 2022 Dec 2;14(23):5965. doi: 10.3390/cancers14235965 (PMC9738162; doi:10.3390/cancers14235965)
Supplement: Supplementary file 1 [file cancers-14-05965-s001.zip › cancers-2040851-supplementary.pdf]

Supplementary Data; Text, Tables and Figures

Supplementary Text

Methods S1

Copy number profile smoothing for CCNE1 classification

First, a baseline, unsmoothed segmentation was performed using CBS. This however produced several noisy profiles, identified by expert classification. Next, using logistic regression, the number of segments in the copy number profile (N_s) and the observed read variance $(\widehat{\sigma^2})$were determined to be most predictive of being classified as noisy in the previous step. Using these variables, a boolean smoothing function, $Sm(N_s, \widehat{\sigma^2})$, was defined with: $Sm(N_s, \widehat{\sigma^2}) = (N_s > t_{N_s})$ & $(\widehat{\sigma^2} > t_{\widehat{\sigma^2}})$. N_s represents the number of segments and $\widehat{\sigma^2}$ the observed variance of the reads at the currently used smoothing parameter. t_{N_s} and $t_{\widehat{\sigma^2}}$ represent threshold parameters which are user selected. If both the number of segments and the variance exceed their respective thresholds, $Sm(N_s, \widehat{\sigma^2})$ evaluates to True and the segmentation is smoothed further. This process is done iteratively until $Sm(N_s, \widehat{\sigma^2})$ evaluates to False for all cases. The final segmentation used for each sample is the least-smoothed segmentation for which $Sm(N_s, \widehat{\sigma^2})$ still returns False. t_{N_s} and $t_{\widehat{\sigma^2}}$ were set at 400 and 0.12 respectively. The baseline segmentation used standard CBS settings with the addition of undoing splits which were not at least 1.5 standard deviations apart. Segmentations were smoothed by iteratively increasing the minimal number of standard deviations required for a split.

Supplementary Tables

Table S1. Median overall survival of immune cell densities per molecular subtype

	BRCAm profile	non-BRCAmut HRD	CCNE1 amplification	Double classifier	NSMP
**CD8**					
≤100	58.7 (30-109)	**40.4 (25-69)***	26.8 (16-50)	26.1 (18-58)	33.2 (15-53)
>100	48.6 (36-97)	**44.2 (27-X)***	36.1 (18-51)	48.6 (19-62)	37.5 (20-82)
**CD20**					
≤50	58.2 (30-99)	**40.0 (23-70)***	26.8 (16-51)	26.1 (19-62)	35.4 (18-56)
> 50	44.0 (25-97)	**67.8 (41-X)***	36.1 (36-X)	47.2 (18-83)	37.2 (20-77)
**CD68**					
≤100	46.1 (36-86)	40.4 (26-72)	25.8 (16-51)	27.0 (18-52)	33.4 (18-56)
>100	57.7 (27-110)	44.2 (19-85)	36.1 (26-48)	55.6 (19-75)	37.5 (25-84)
**CD103**					
≤100	46.1 (29-97)	40.4 (23-72)	26.8 (18-50)	30.0 (19-56)	**33.4 (16-55)***
>100	60.4 (41-110)	44.2 (38-105)	45.4 (14-57)	52.1 (18-66)	**62.9 (19-X)***

*P < 0.05

Abbreviations: BRCAm profile: BRCA mutation or BRCA1 promotor methylation; HRD: homologous repair deficient; Double classifier: non-BRCAmut HRD and CCNE1 gain/amplification; NSMP: no specific molecular profile.

Table S2. Progression-free survival analysis, immune cell composition

	**Crude HR (95% CI)**	**Adjusted* HR (95% CI)**
**CD8**		
≤100	REF	REF
>100	**0.74 (0.56-0.98)**	0.83 (0.62-1.10)
**CD20**		
≤50	REF	REF
> 50	**0.64 (0.45-0.91)**	**0.56 (0.40-0.80)**
**CD68**		
≤100	REF	REF
>100	**0.68 (0.51-0.90)**	0.98 (0.72-1.32)
**CD103**		
≤100	REF	REF
>100	**0.59 (0.43-0.83)**	0.72 (0.52-1.01)

*Data was adjusted for age, FIGO stage, therapy sequence and outcome of surgery

Abbreviations: CI: Confidence interval; HR: Hazard ratio; REF: reference.

Supplementary Figures

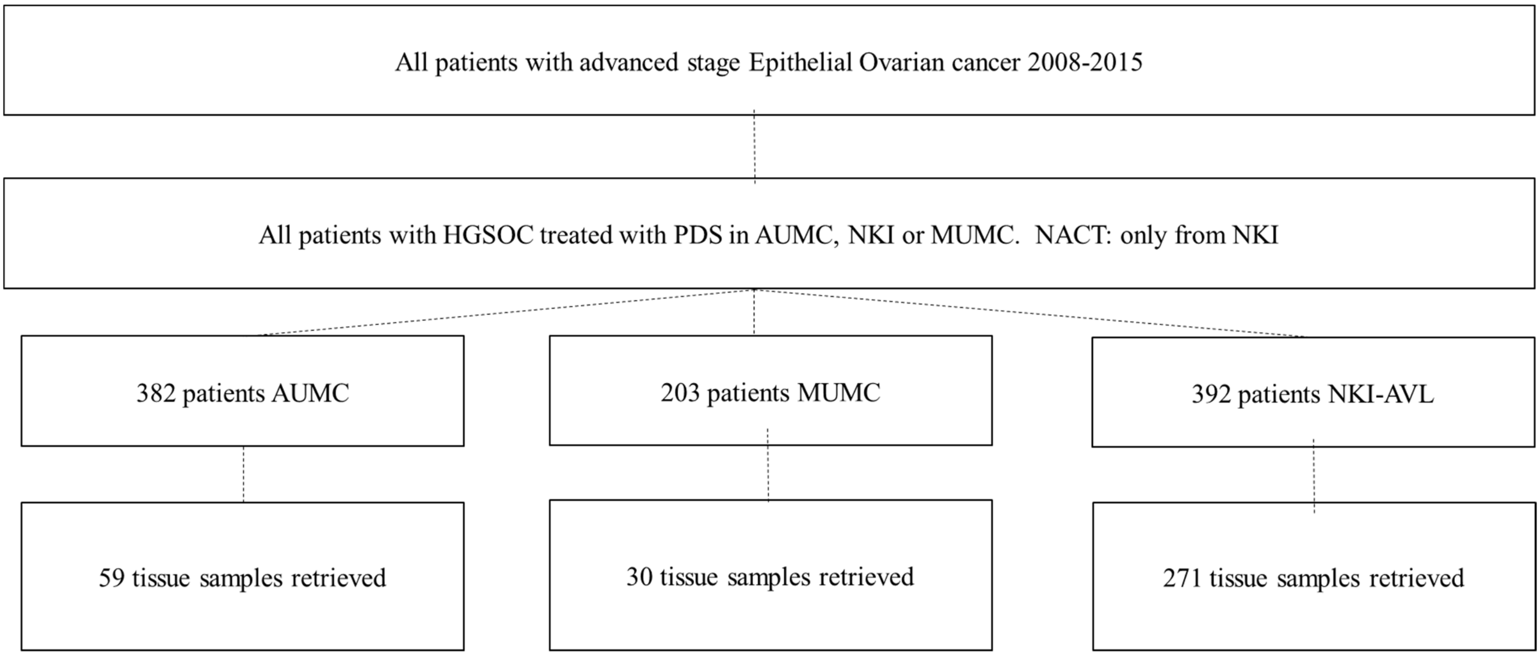

Figure S1. Flowchart of patient selection and tissue retrieval

Abbreviations: NKI-AVL: Netherlands Cancer Institute - Antoni van Leeuwenhoek Hospital; MUMC: Maastricht University Medical Centre (MUMC); AUMC: Amsterdam University Medical Centre; PDS: primary debulking surgery; NACT: neoadjuvant chemotherapy

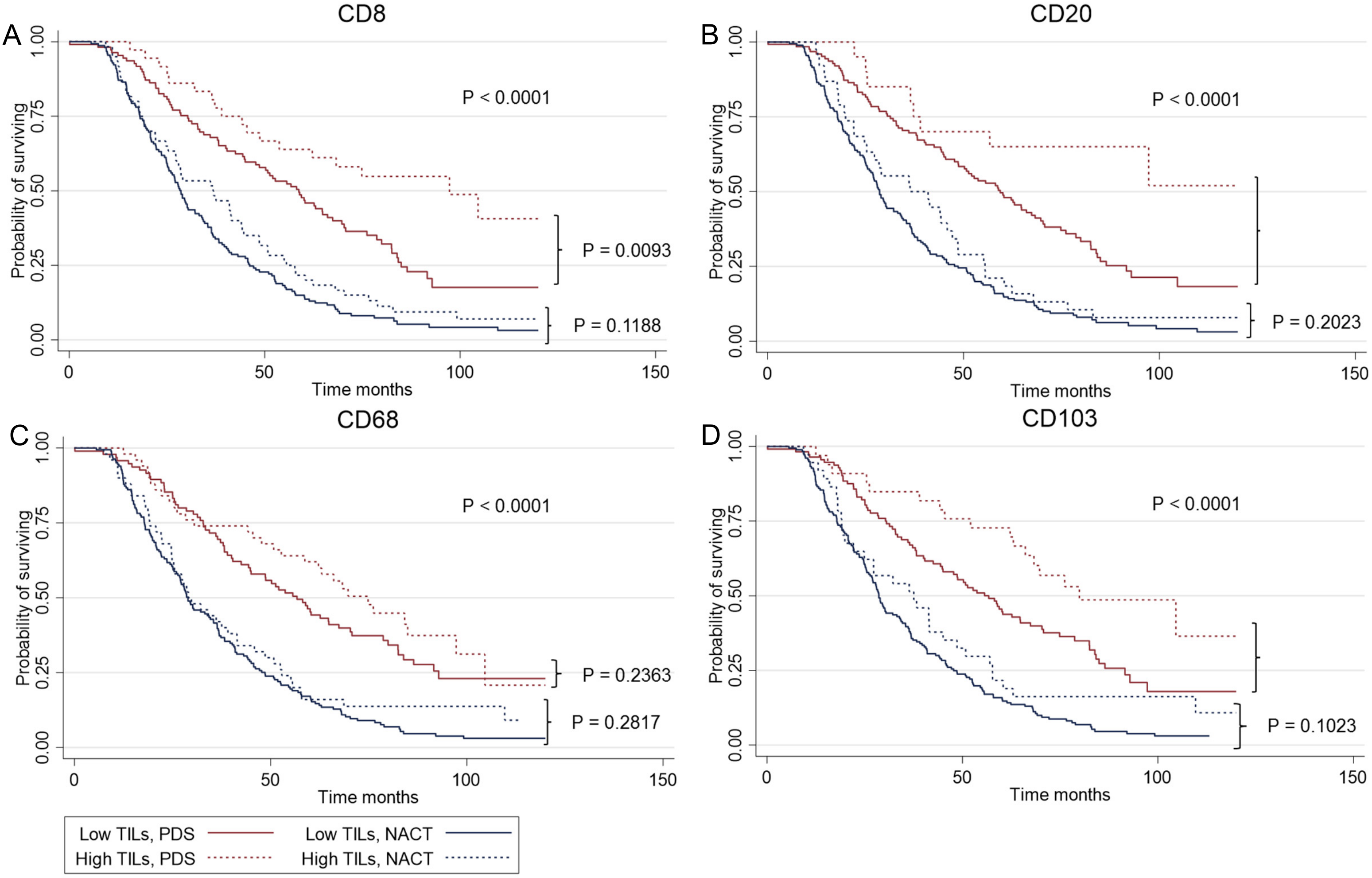

Figure S2. Survival analyses of patients with HGSOC, according to immune cell densities and therapy sequence.

Panels A to D show Kaplan–Meier curves for overall survival according to Tumor infiltrating lymphocyte (TIL) densities (high; dotted line, low; solid) subgrouped by therapy sequence (Neoadjuvant chemotherapy; blue, Primary debulking: maroon) of CD8 cells (A), CD20 (B), CD68 (C) and CD103 (D) respectively. P values were derived with the use of the log-rank statistic depicting the P value of the entire Kaplan Meier and between TIL densities within the same treatment sequence (PDS or NACT).

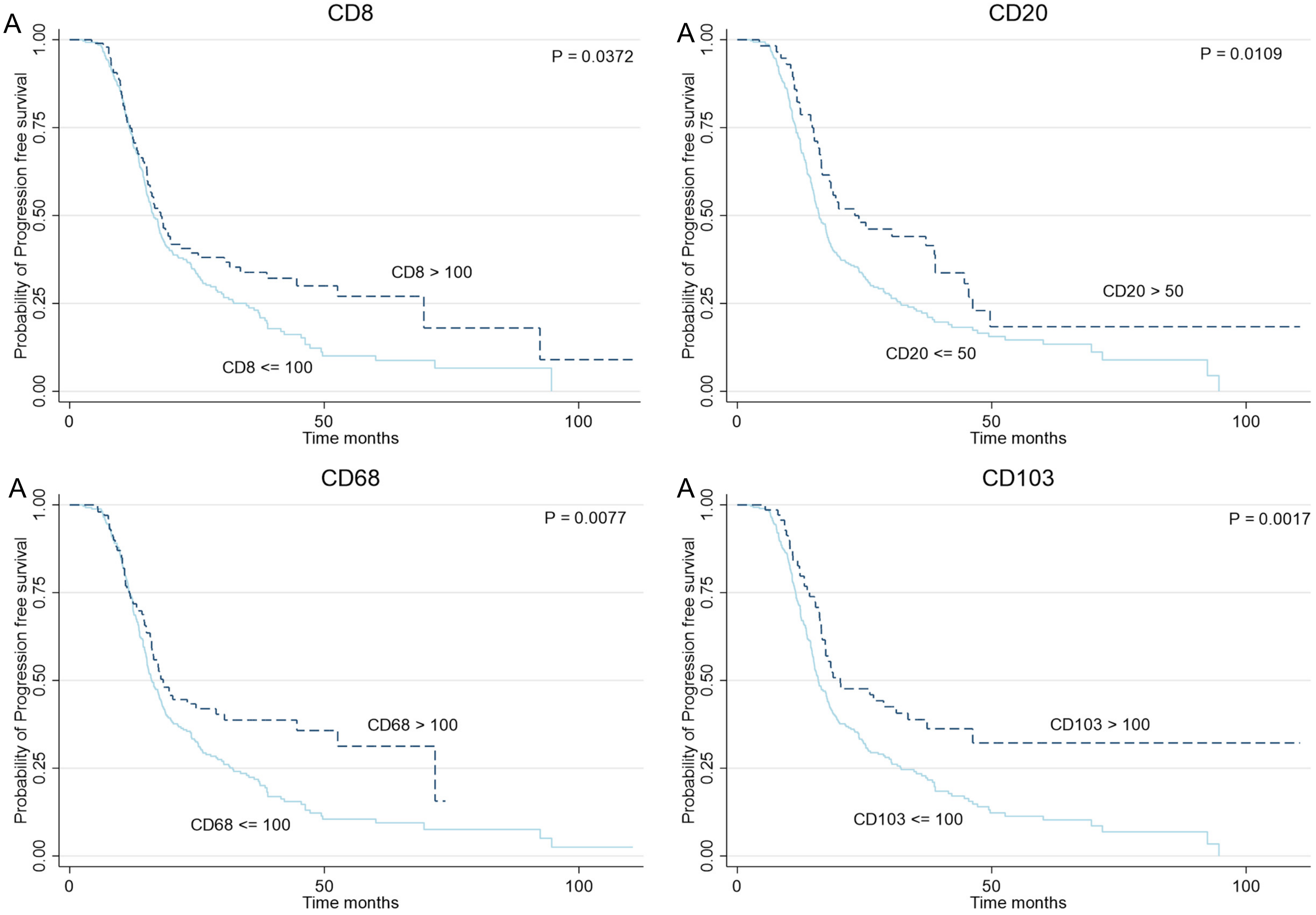

Figure S3. Survival Analyses of Patients with HGSOC, According to immune cell densities.

Panels A to D show Kaplan–Meier curves for progression free survival according to density of CD8 cells (A), CD20 (B), CD68 (C) and CD103 (D) respectively. P values were derived with the use of the log-rank statistic.
